# (4*R*,7*S*)-2-Amino-4-(3,4-dimeth­oxy­phen­yl)-5-oxo-7-phenyl-5,6,7,8-tetra­hydro-4*H*-chromene-3-carbonitrile monohydrate

**DOI:** 10.1107/S1600536811056285

**Published:** 2012-01-14

**Authors:** Rong Sun, Dong-Dong Wu, Ke Wang, Wei Huang, Yang-Bing Ou

**Affiliations:** a Shandong Academy of Chinese Medicine, Jinan 250014, People’s Republic of China; b Postdoctoral Research Station of Shandong University of TCM, Jinan 250355, People’s Republic of China; c Shanghai Institute of Materia Medica, Chinese Academy of Sciences, Shanghai 201203, People’s Republic of China; d Key Laboratory of Nuclear Medicine, Ministry of Health, Jiangsu Key Laboratory of Molecular Nuclear Medicine, Jiangsu Institute of Nuclear Medicine, Wuxi 214063, People’s Republic of China

## Abstract

The title compound, C_24_H_22_N_2_O_4_·H_2_O, was obtained by the reaction of 3,4-dimeth­oxy­benzaldehyde, malononitrile and 5-phenyl­cyclo­hexane-1,3-dione. The cyclo­hexyl and pyran rings show half-boat and V-shaped conformations, respectively. The dihedral angle between the phenyl and benzene ring planes is 30.67 (9)°. The organic mol­ecules are packed in a two-dimensional network parallel to the *bc* plane stabilized by inter­molecular N—H⋯N and N—H⋯O hydrogen bonds.

## Related literature

For background to 4-aryl-4*H*-chromene and its derivatives, see: Kemnitzer *et al.* (2004 ▶, 2005 ▶, 2007 ▶, 2008 ▶); Gourdeau *et al.* (2004 ▶); Foroumadi *et al.* (2007 ▶); Mahdavi *et al.* (2011 ▶). For the synthesis of 4-aryl-4*H*-chromene and its derivatives, see: Wen *et al.* (2006 ▶); Kidwai *et al.* (2005 ▶); Yadav *et al.* (2009 ▶); Li *et al.* (2008 ▶). For related compounds, see: Gourdeau *et al.* (2004 ▶); Foroumadi *et al.* (2007 ▶).
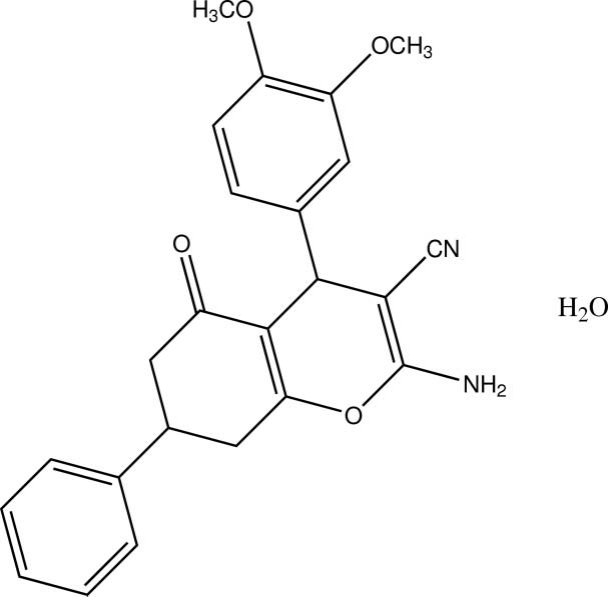


## Experimental

### 

#### Crystal data

C_24_H_22_N_2_O_4_·H_2_O
*M* *_r_* = 420.45
Monoclinic, 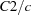

*a* = 29.008 (16) Å
*b* = 16.146 (8) Å
*c* = 12.068 (6) Å
β = 110.486 (9)°
*V* = 5295 (5) Å^3^
*Z* = 8
Mo *K*α radiation
μ = 0.07 mm^−1^
*T* = 291 K
0.38 × 0.32 × 0.24 mm

#### Data collection

Bruker SMART APEX CCD diffractometer
Absorption correction: multi-scan (*SADABS*; Bruker, 2000 ▶) *T*_min_ = 0.972, *T*_max_ = 0.982
14295 measured reflections
5212 independent reflections
3266 reflections with *I* > 2σ(*I*)
*R*_int_ = 0.041

#### Refinement

*R*[*F* ^2^ > 2σ(*F* ^2^)] = 0.058
*wR*(*F* ^2^) = 0.132
*S* = 1.00
5212 reflections
305 parameters
H-atom parameters constrained
Δρ_max_ = 0.16 e Å^−3^
Δρ_min_ = −0.15 e Å^−3^

### 

Data collection: *SMART* (Bruker, 2000 ▶); cell refinement: *SAINT* (Bruker, 2000 ▶); data reduction: *SAINT*; program(s) used to solve structure: *SHELXTL* (Sheldrick, 2008 ▶); program(s) used to refine structure: *SHELXTL*; molecular graphics: *SHELXTL*; software used to prepare material for publication: *SHELXTL*.

## Supplementary Material

Crystal structure: contains datablock(s) I, global. DOI: 10.1107/S1600536811056285/bx2390sup1.cif

Structure factors: contains datablock(s) I. DOI: 10.1107/S1600536811056285/bx2390Isup2.hkl

Supplementary material file. DOI: 10.1107/S1600536811056285/bx2390Isup3.cml

Additional supplementary materials:  crystallographic information; 3D view; checkCIF report

## Figures and Tables

**Table 1:** Hydrogen-bond geometry (Å, °)

*D*—H⋯*A*	*D*—H	H⋯*A*	*D*⋯*A*	*D*—H⋯*A*
N1—H1*A*⋯N2^i^	0.86	2.20	3.042 (3)	167
N1—H1*B*⋯O2^ii^	0.86	2.12	2.935 (3)	158

Symmetry codes: (i) 
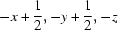
; (ii) 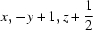.

**Fig. 1.**
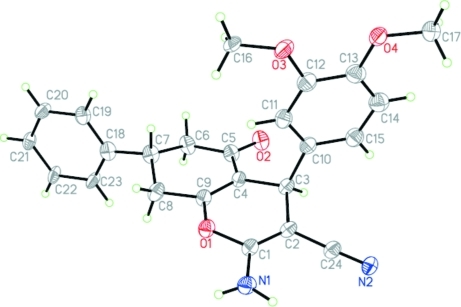
The molecular structure of the title compound, showing 30% probability displacement and the atom numbering scheme. The water molecule is omitted by clarity.

## supplementary crystallographic information

### Comment

4-Aryl-4*H*-chromene compounds are a very important series of chromene derivatives, because they can be introduced as potent apoptosis inducing agents and exhibit anticancer activities (Kemnitzer *et al.*, 2004, 2005, 2007, 2008; Gourdeau *et al.*, 2004; Foroumadi *et al.*, 2007; Mahdavi *et al.*, 2011). Herein,we report the synthesis and crystal structure of a new 4-Aryl-4*H*-chromene derivative.The molecular structure of the title compound is shown in Fig.1. In structure of the title compound, the cyclohexyl ring shows in a half-boat conformation and the pyran ring shows in "V" shape. The dihedral angle between phenyl and benzene rings planes is 30.67 (9)°. The organic molecules are packing in a bi-dimensional network stabilized by intermolecular N—H···N, N—H···O hydrogen bonds, Table1.

### Experimental

The title compound was synthesized using methods described by Wen *et al.* 2006 & Kidwai *et al.* 2005. Single crystals of the title compound suitable for X-ray analysis were obtained by evaporating the solution of compound in ethanol at room temperature for one week.

### Refinement

All H atoms were fixed geometrically and treated as riding with C—H = 0.96 Å (methoxyl), 0.93 Å (phenyl), N–H = 0.86 Å (amino), O–H = 0.85 Å (water) and 0.97–0.98 Å (methylene), with *U*_iso_(H) = 1.2*U*_eq_ (phenyl) or *U*_iso_(H) = 1.5*U*_eq_ (methoxyl, NH and OH).

### Crystal data

**Table:**

C_24_H_22_N_2_O_4_·H_2_O	*F*(000) = 1776
*M**_r_* = 420.45	*D*_x_ = 1.055 Mg m^−^^3^
Monoclinic, *C*2/*c*	Mo *K*α radiation, λ = 0.71073 Å
Hall symbol: -C 2yc	Cell parameters from 2449 reflections
*a* = 29.008 (16) Å	θ = 2.5–21.9°
*b* = 16.146 (8) Å	µ = 0.07 mm^−^^1^
*c* = 12.068 (6) Å	*T* = 291 K
β = 110.486 (9)°	Block, colorless
*V* = 5295 (5) Å^3^	0.38 × 0.32 × 0.24 mm
*Z* = 8	

### Data collection

**Table:**

Bruker SMART APEX CCD diffractometer	5212 independent reflections
Radiation source: sealed tube	3266 reflections with *I* > 2σ(*I*)
graphite	*R*_int_ = 0.041
φ and ω scans	θ_max_ = 26.0°, θ_min_ = 1.5°
Absorption correction: multi-scan (*SADABS*; Bruker, 2000)	*h* = −35→28
*T*_min_ = 0.972, *T*_max_ = 0.982	*k* = −16→19
14295 measured reflections	*l* = −14→14

### Refinement

**Table:**

Refinement on *F*^2^	Primary atom site location: structure-invariant direct methods
Least-squares matrix: full	Secondary atom site location: difference Fourier map
*R*[*F*^2^ > 2σ(*F*^2^)] = 0.058	Hydrogen site location: inferred from neighbouring sites
*wR*(*F*^2^) = 0.132	H-atom parameters constrained
*S* = 1.00	*w* = 1/[σ^2^(*F*_o_^2^) + (0.0629*P*)^2^] where *P* = (*F*_o_^2^ + 2*F*_c_^2^)/3
5212 reflections	(Δ/σ)_max_ < 0.001
305 parameters	Δρ_max_ = 0.16 e Å^−^^3^
0 restraints	Δρ_min_ = −0.15 e Å^−^^3^

### Special details

**Table:**

Experimental. Least-squares planes (*x*,*y*,*z* in crystal coordinates) and deviations from them (* indicates atom used to define plane)18.7999 (0.0253) *x* + 6.0274 (0.0140) *y* + 4.7668 (0.0109) *z* = 7.5153 (0.0082)* 0.0227 (0.0013) C4 * -0.0288 (0.0015) C5 * 0.0178 (0.0011) C6 * -0.0068 (0.0011) C8 * -0.0049 (0.0014) C9Rms deviation of fitted atoms = 0.0186- 0.7360 (0.0244) *x* + 15.4410 (0.0094) *y* - 3.1844 (0.0102) *z* = 8.2455 (0.0098)Angle to previous plane (with approximate e.s.d.) = 81.53 (8)* 0.0000 (0.0014) C10 * 0.0000 (0.0014) C11 * 0.0000 (0.0015) C12 * 0.0000 (0.0015) C13 * 0.0000 (0.0015) C14 * 0.0000 (0.0015) C15Rms deviation of fitted atoms = 0.00006.5144 (0.0251) *x* + 11.2780 (0.0120) *y* - 8.6296 (0.0089) *z* = 7.8493 (0.0106)Angle to previous plane (with approximate e.s.d.) = 30.67 (9)* 0.0000 (0.0015) C18 * 0.0000 (0.0015) C19 * 0.0000 (0.0015) C20 * 0.0000 (0.0015) C21 * 0.0000 (0.0015) C22 * 0.0000 (0.0016) C23Rms deviation of fitted atoms = 0.0000
Geometry. All e.s.d.'s (except the e.s.d. in the dihedral angle between two l.s. planes) are estimated using the full covariance matrix. The cell e.s.d.'s are taken into account individually in the estimation of e.s.d.'s in distances, angles and torsion angles; correlations between e.s.d.'s in cell parameters are only used when they are defined by crystal symmetry. An approximate (isotropic) treatment of cell e.s.d.'s is used for estimating e.s.d.'s involving l.s. planes.
Refinement. Refinement of *F*^2^ against ALL reflections. The weighted *R*-factor *wR* and goodness of fit *S* are based on *F*^2^, conventional *R*-factors *R* are based on *F*, with *F* set to zero for negative *F*^2^. The threshold expression of *F*^2^ > σ(*F*^2^) is used only for calculating *R*-factors(gt) *etc*. and is not relevant to the choice of reflections for refinement. *R*-factors based on *F*^2^ are statistically about twice as large as those based on *F*, and *R*- factors based on ALL data will be even larger.

### Fractional atomic coordinates and isotropic or equivalent isotropic displacement parameters (Å^2^)

**Table:**

	*x*	*y*	*z*	*U*_iso_*/*U*_eq_	Occ. (<1)
C1	0.21676 (7)	0.42190 (12)	0.10641 (17)	0.0398 (4)	
C2	0.23258 (7)	0.43944 (11)	0.01610 (16)	0.0384 (4)	
C3	0.23849 (7)	0.52781 (11)	−0.02193 (17)	0.0380 (4)	
H3A	0.2253	0.5299	−0.1086	0.046*	
C4	0.20768 (7)	0.58404 (12)	0.02376 (16)	0.0363 (4)	
C5	0.19297 (7)	0.66544 (12)	−0.03195 (18)	0.0426 (5)	
C6	0.16490 (8)	0.72255 (13)	0.01636 (17)	0.0467 (5)	
H6A	0.1315	0.7258	−0.0399	0.056*	
H6B	0.1793	0.7773	0.0216	0.056*	
C7	0.16233 (7)	0.70135 (12)	0.13490 (18)	0.0432 (5)	
H7A	0.1946	0.7191	0.1892	0.052*	
C8	0.16176 (8)	0.61261 (13)	0.16256 (19)	0.0480 (5)	
H8A	0.1280	0.5929	0.1333	0.058*	
H8B	0.1744	0.6055	0.2478	0.058*	
C9	0.19168 (7)	0.56133 (12)	0.10981 (16)	0.0378 (4)	
C10	0.29243 (7)	0.55177 (11)	0.01855 (18)	0.0396 (4)	
C11	0.31728 (7)	0.57684 (13)	0.13438 (17)	0.0443 (5)	
H11A	0.3000	0.5865	0.1850	0.053*	
C12	0.36802 (8)	0.58754 (13)	0.17454 (19)	0.0473 (5)	
C13	0.39391 (8)	0.57317 (13)	0.0989 (2)	0.0505 (5)	
C14	0.36906 (8)	0.54809 (14)	−0.0170 (2)	0.0544 (6)	
H14A	0.3864	0.5385	−0.0676	0.065*	
C15	0.31832 (8)	0.53739 (13)	−0.05712 (19)	0.0484 (5)	
H15A	0.3017	0.5206	−0.1346	0.058*	
C16	0.37141 (7)	0.65844 (12)	0.35114 (17)	0.0418 (5)	
H16A	0.3953	0.6784	0.4234	0.063*	
H16B	0.3489	0.6218	0.3691	0.063*	
H16C	0.3536	0.7044	0.3056	0.063*	
C17	0.47282 (7)	0.55420 (13)	0.08253 (18)	0.0442 (5)	
H17A	0.5070	0.5570	0.1308	0.066*	
H17B	0.4667	0.5891	0.0145	0.066*	
H17C	0.4643	0.4981	0.0574	0.066*	
C18	0.12798 (8)	0.75372 (13)	0.17207 (18)	0.0465 (5)	
C19	0.15023 (8)	0.80989 (13)	0.26227 (18)	0.0444 (5)	
H19A	0.1843	0.8140	0.2934	0.053*	
C20	0.12148 (7)	0.85989 (12)	0.30591 (19)	0.0453 (5)	
H20A	0.1364	0.8975	0.3663	0.054*	
C21	0.07048 (7)	0.85373 (12)	0.25936 (17)	0.0425 (5)	
H21C	0.0512	0.8872	0.2886	0.051*	
C22	0.04824 (8)	0.79756 (12)	0.16916 (18)	0.0448 (5)	
H22A	0.0141	0.7934	0.1380	0.054*	
C23	0.07698 (7)	0.74756 (12)	0.12551 (18)	0.0459 (5)	
H23A	0.0621	0.7100	0.0652	0.055*	
C24	0.24929 (7)	0.37408 (12)	−0.03676 (18)	0.0424 (5)	
N1	0.21337 (6)	0.34897 (10)	0.15312 (15)	0.0438 (4)	
H1A	0.2221	0.3049	0.1256	0.053*	
H1B	0.2025	0.3455	0.2108	0.053*	
N2	0.26326 (6)	0.32244 (10)	−0.08241 (14)	0.0418 (4)	
O1	0.20008 (5)	0.48364 (8)	0.16179 (11)	0.0403 (3)	
O2	0.20311 (5)	0.68522 (9)	−0.11773 (12)	0.0473 (4)	
O3	0.39552 (5)	0.61541 (9)	0.28562 (13)	0.0495 (4)	
O4	0.44392 (5)	0.58132 (9)	0.14876 (12)	0.0461 (4)	
O1W	1.0000	0.7117 (3)	0.7500	0.0600 (13)	0.50
H1X	1.0148	0.6920	0.7062	0.072*	0.25
H1Y	0.9771	0.7439	0.7095	0.072*	0.25
O2W	0.0778 (2)	0.4029 (3)	0.9183 (5)	0.0473 (14)	0.25
H2X	0.0654	0.3666	0.8652	0.057*	0.25
H2Y	0.1088	0.4040	0.9360	0.057*	0.25
O3W	0.0547 (2)	0.5322 (3)	0.9458 (5)	0.0514 (15)	0.25
H3X	0.0710	0.5671	0.9215	0.062*	0.25
H3Y	0.0242	0.5384	0.9073	0.062*	0.25
O6W	0.0707 (2)	0.4226 (4)	0.1806 (5)	0.0497 (14)	0.25
H6X	0.0635	0.3823	0.1323	0.060*	0.25
H6Y	0.0502	0.4617	0.1534	0.060*	0.25

### Atomic displacement parameters (Å^2^)

**Table:**

	*U*^11^	*U*^22^	*U*^33^	*U*^12^	*U*^13^	*U*^23^
C1	0.0460 (10)	0.0365 (10)	0.0360 (10)	0.0014 (8)	0.0133 (9)	−0.0021 (8)
C2	0.0469 (10)	0.0295 (10)	0.0356 (10)	0.0015 (8)	0.0104 (8)	−0.0028 (8)
C3	0.0469 (11)	0.0321 (10)	0.0340 (10)	0.0026 (8)	0.0128 (8)	−0.0016 (7)
C4	0.0419 (10)	0.0344 (10)	0.0347 (9)	0.0029 (7)	0.0160 (8)	−0.0021 (7)
C5	0.0405 (11)	0.0430 (11)	0.0445 (11)	0.0090 (8)	0.0151 (8)	0.0079 (9)
C6	0.0482 (11)	0.0503 (12)	0.0446 (11)	0.0153 (10)	0.0201 (9)	0.0186 (10)
C7	0.0422 (11)	0.0415 (11)	0.0448 (11)	0.0168 (8)	0.0139 (8)	0.0143 (9)
C8	0.0563 (12)	0.0423 (11)	0.0430 (11)	0.0111 (10)	0.0143 (10)	−0.0020 (9)
C9	0.0457 (10)	0.0342 (10)	0.0344 (10)	0.0055 (8)	0.0150 (8)	0.0038 (8)
C10	0.0444 (10)	0.0297 (9)	0.0435 (11)	0.0058 (8)	0.0137 (8)	0.0020 (8)
C11	0.0492 (12)	0.0476 (11)	0.0361 (10)	0.0030 (9)	0.0148 (9)	0.0001 (9)
C12	0.0493 (12)	0.0464 (12)	0.0418 (11)	0.0014 (9)	0.0103 (9)	−0.0018 (9)
C13	0.0478 (12)	0.0436 (12)	0.0585 (13)	0.0009 (9)	0.0165 (10)	0.0011 (10)
C14	0.0521 (13)	0.0503 (13)	0.0574 (14)	0.0013 (10)	0.0150 (10)	−0.0005 (11)
C15	0.0556 (13)	0.0463 (12)	0.0424 (12)	0.0019 (10)	0.0158 (10)	−0.0002 (9)
C16	0.0407 (11)	0.0431 (11)	0.0379 (10)	−0.0079 (9)	0.0092 (8)	−0.0167 (9)
C17	0.0443 (11)	0.0435 (11)	0.0435 (11)	0.0085 (9)	0.0135 (8)	0.0054 (9)
C18	0.0489 (11)	0.0468 (12)	0.0452 (11)	0.0134 (9)	0.0181 (9)	0.0103 (9)
C19	0.0398 (10)	0.0470 (12)	0.0472 (11)	0.0114 (9)	0.0164 (8)	0.0038 (10)
C20	0.0440 (11)	0.0395 (11)	0.0494 (12)	0.0021 (9)	0.0128 (9)	−0.0149 (9)
C21	0.0437 (12)	0.0444 (12)	0.0411 (11)	0.0141 (9)	0.0172 (9)	−0.0048 (9)
C22	0.0438 (10)	0.0490 (12)	0.0429 (10)	0.0120 (9)	0.0170 (8)	−0.0163 (9)
C23	0.0494 (12)	0.0411 (10)	0.0467 (11)	0.0047 (8)	0.0160 (9)	−0.0200 (9)
C24	0.0502 (11)	0.0358 (10)	0.0411 (11)	0.0023 (9)	0.0156 (9)	−0.0006 (9)
N1	0.0407 (9)	0.0338 (9)	0.0551 (11)	0.0082 (7)	0.0145 (8)	0.0036 (8)
N2	0.0461 (10)	0.0416 (9)	0.0376 (9)	0.0023 (7)	0.0148 (7)	−0.0069 (7)
O1	0.0436 (7)	0.0341 (7)	0.0434 (8)	0.0108 (6)	0.0156 (6)	0.0040 (6)
O2	0.0504 (8)	0.0517 (9)	0.0410 (7)	0.0151 (7)	0.0176 (6)	0.0185 (7)
O3	0.0513 (8)	0.0417 (8)	0.0515 (9)	0.0009 (6)	0.0131 (7)	−0.0146 (7)
O4	0.0443 (8)	0.0452 (8)	0.0474 (8)	0.0000 (6)	0.0143 (6)	−0.0122 (7)
O1W	0.054 (2)	0.052 (3)	0.048 (2)	0.000	−0.0156 (19)	0.000
O2W	0.043 (3)	0.055 (4)	0.043 (3)	−0.021 (3)	0.014 (2)	−0.011 (3)
O3W	0.049 (3)	0.053 (3)	0.050 (3)	−0.016 (3)	0.013 (3)	−0.021 (3)
O6W	0.051 (3)	0.045 (3)	0.048 (3)	0.018 (3)	0.010 (3)	0.004 (3)

### Geometric parameters (Å, °)

**Table:**

C1—N1	1.324 (3)	C15—H15A	0.9300
C1—C2	1.352 (3)	C16—O3	1.409 (2)
C1—O1	1.379 (2)	C16—H16A	0.9600
C2—C24	1.404 (3)	C16—H16B	0.9600
C2—C3	1.527 (3)	C16—H16C	0.9600
C3—C4	1.507 (3)	C17—O4	1.415 (3)
C3—C10	1.517 (3)	C17—H17A	0.9600
C3—H3A	0.9800	C17—H17B	0.9600
C4—C9	1.329 (3)	C17—H17C	0.9600
C4—C5	1.470 (3)	C18—C19	1.390 (3)
C5—O2	1.214 (3)	C18—C23	1.390 (3)
C5—C6	1.478 (3)	C19—C20	1.390 (3)
C6—C7	1.498 (3)	C19—H19A	0.9300
C6—H6A	0.9700	C20—C21	1.390 (3)
C6—H6B	0.9700	C20—H20A	0.9300
C7—C8	1.473 (3)	C21—C22	1.390 (3)
C7—C18	1.491 (3)	C21—H21C	0.9300
C7—H7A	0.9800	C22—C23	1.390 (3)
C8—C9	1.494 (3)	C22—H22A	0.9300
C8—H8A	0.9700	C23—H23A	0.9300
C8—H8B	0.9700	C24—N2	1.149 (3)
C9—O1	1.385 (2)	N1—H1A	0.8601
C10—C11	1.390 (3)	N1—H1B	0.8607
C10—C15	1.390 (3)	O1W—H1X	0.8500
C11—C12	1.390 (3)	O1W—H1Y	0.8500
C11—H11A	0.9300	O2W—H2X	0.8501
C12—O3	1.374 (2)	O2W—H2Y	0.8499
C12—C13	1.390 (3)	O3W—H3X	0.8499
C13—O4	1.369 (3)	O3W—H3Y	0.8500
C13—C14	1.390 (3)	O6W—H6X	0.8500
C14—C15	1.390 (3)	O6W—H6Y	0.8501
C14—H14A	0.9300		
			
N1—C1—C2	128.76 (18)	O4—C13—C14	124.6 (2)
N1—C1—O1	110.24 (18)	C12—C13—C14	120.0 (2)
C2—C1—O1	120.99 (18)	C15—C14—C13	120.0 (2)
C1—C2—C24	118.50 (18)	C15—C14—H14A	120.0
C1—C2—C3	122.92 (17)	C13—C14—H14A	120.0
C24—C2—C3	118.22 (18)	C14—C15—C10	120.0 (2)
C4—C3—C10	113.91 (15)	C14—C15—H15A	120.0
C4—C3—C2	108.04 (17)	C10—C15—H15A	120.0
C10—C3—C2	110.68 (15)	O3—C16—H16A	109.5
C4—C3—H3A	108.0	O3—C16—H16B	109.5
C10—C3—H3A	108.0	H16A—C16—H16B	109.5
C2—C3—H3A	108.0	O3—C16—H16C	109.5
C9—C4—C5	118.64 (18)	H16A—C16—H16C	109.5
C9—C4—C3	122.25 (18)	H16B—C16—H16C	109.5
C5—C4—C3	119.06 (17)	O4—C17—H17A	109.5
O2—C5—C4	120.47 (18)	O4—C17—H17B	109.5
O2—C5—C6	120.24 (18)	H17A—C17—H17B	109.5
C4—C5—C6	119.28 (18)	O4—C17—H17C	109.5
C5—C6—C7	116.90 (16)	H17A—C17—H17C	109.5
C5—C6—H6A	108.1	H17B—C17—H17C	109.5
C7—C6—H6A	108.1	C19—C18—C23	120.00 (19)
C5—C6—H6B	108.1	C19—C18—C7	115.45 (19)
C7—C6—H6B	108.1	C23—C18—C7	124.5 (2)
H6A—C6—H6B	107.3	C20—C19—C18	120.0 (2)
C8—C7—C18	114.96 (19)	C20—C19—H19A	120.0
C8—C7—C6	116.55 (18)	C18—C19—H19A	120.0
C18—C7—C6	114.81 (16)	C19—C20—C21	120.00 (19)
C8—C7—H7A	102.5	C19—C20—H20A	120.0
C18—C7—H7A	102.5	C21—C20—H20A	120.0
C6—C7—H7A	102.5	C22—C21—C20	120.00 (18)
C7—C8—C9	112.86 (19)	C22—C21—H21C	120.0
C7—C8—H8A	109.0	C20—C21—H21C	120.0
C9—C8—H8A	109.0	C21—C22—C23	120.0 (2)
C7—C8—H8B	109.0	C21—C22—H22A	120.0
C9—C8—H8B	109.0	C23—C22—H22A	120.0
H8A—C8—H8B	107.8	C22—C23—C18	120.00 (19)
C4—C9—O1	123.35 (18)	C22—C23—H23A	120.0
C4—C9—C8	126.45 (18)	C18—C23—H23A	120.0
O1—C9—C8	110.19 (16)	N2—C24—C2	177.8 (2)
C11—C10—C15	120.00 (19)	C1—N1—H1A	119.7
C11—C10—C3	120.77 (19)	C1—N1—H1B	120.3
C15—C10—C3	118.65 (18)	H1A—N1—H1B	119.9
C12—C11—C10	120.0 (2)	C1—O1—C9	118.10 (15)
C12—C11—H11A	120.0	C12—O3—C16	118.45 (17)
C10—C11—H11A	120.0	C13—O4—C17	117.90 (16)
O3—C12—C11	124.3 (2)	H1X—O1W—H1Y	109.5
O3—C12—C13	115.7 (2)	H2X—O2W—H2Y	109.5
C11—C12—C13	120.0 (2)	H3X—O3W—H3Y	109.5
O4—C13—C12	115.35 (19)	H6X—O6W—H6Y	109.5
			
N1—C1—C2—C24	−1.8 (3)	C10—C11—C12—O3	177.15 (19)
O1—C1—C2—C24	179.83 (17)	C10—C11—C12—C13	0.0 (3)
N1—C1—C2—C3	−174.77 (19)	O3—C12—C13—O4	5.8 (3)
O1—C1—C2—C3	6.8 (3)	C11—C12—C13—O4	−176.76 (18)
C1—C2—C3—C4	−20.2 (2)	O3—C12—C13—C14	−177.39 (18)
C24—C2—C3—C4	166.75 (16)	C11—C12—C13—C14	0.0 (3)
C1—C2—C3—C10	105.1 (2)	O4—C13—C14—C15	176.4 (2)
C24—C2—C3—C10	−67.9 (2)	C12—C13—C14—C15	0.0 (3)
C10—C3—C4—C9	−105.4 (2)	C13—C14—C15—C10	0.0 (3)
C2—C3—C4—C9	18.0 (2)	C11—C10—C15—C14	0.0 (3)
C10—C3—C4—C5	77.3 (2)	C3—C10—C15—C14	−171.30 (18)
C2—C3—C4—C5	−159.31 (16)	C8—C7—C18—C19	112.6 (2)
C9—C4—C5—O2	−173.29 (19)	C6—C7—C18—C19	−108.0 (2)
C3—C4—C5—O2	4.1 (3)	C8—C7—C18—C23	−65.3 (3)
C9—C4—C5—C6	5.6 (3)	C6—C7—C18—C23	74.0 (3)
C3—C4—C5—C6	−176.94 (18)	C23—C18—C19—C20	0.0 (3)
O2—C5—C6—C7	−168.38 (19)	C7—C18—C19—C20	−178.03 (19)
C4—C5—C6—C7	12.7 (3)	C18—C19—C20—C21	0.0 (3)
C5—C6—C7—C8	−33.1 (3)	C19—C20—C21—C22	0.0 (3)
C5—C6—C7—C18	−171.76 (19)	C20—C21—C22—C23	0.0 (3)
C18—C7—C8—C9	172.45 (17)	C21—C22—C23—C18	0.0 (3)
C6—C7—C8—C9	33.8 (3)	C19—C18—C23—C22	0.0 (3)
C5—C4—C9—O1	174.74 (16)	C7—C18—C23—C22	177.85 (19)
C3—C4—C9—O1	−2.6 (3)	C1—C2—C24—N2	−180 (100)
C5—C4—C9—C8	−3.7 (3)	C3—C2—C24—N2	−6(6)
C3—C4—C9—C8	178.96 (18)	N1—C1—O1—C9	−167.40 (15)
C7—C8—C9—C4	−16.4 (3)	C2—C1—O1—C9	11.3 (3)
C7—C8—C9—O1	165.01 (15)	C4—C9—O1—C1	−13.7 (3)
C4—C3—C10—C11	41.8 (2)	C8—C9—O1—C1	165.00 (16)
C2—C3—C10—C11	−80.1 (2)	C11—C12—O3—C16	−19.5 (3)
C4—C3—C10—C15	−146.95 (18)	C13—C12—O3—C16	157.75 (19)
C2—C3—C10—C15	91.1 (2)	C12—C13—O4—C17	169.62 (18)
C15—C10—C11—C12	0.0 (3)	C14—C13—O4—C17	−7.0 (3)
C3—C10—C11—C12	171.12 (17)		

### Hydrogen-bond geometry (Å, °)

**Table:**

*D*—H···*A*	*D*—H	H···*A*	*D*···*A*	*D*—H···*A*
N1—H1A···N2^i^	0.86	2.20	3.042 (3)	167.
N1—H1B···O2^ii^	0.86	2.12	2.935 (3)	158.
.—···..	.	.	.	.

Symmetry codes: (i) −*x*+1/2, −*y*+1/2, −*z*; (ii) *x*, −*y*+1, *z*+1/2.
